# The relationship between the expression of VEGF, EGFR, and HER-2 mRNA in esophageal squamous cell carcinoma (ESCC) and clinicopathological features of different ethnic groups in Xinjiang

**DOI:** 10.1007/s13277-015-3656-z

**Published:** 2015-06-23

**Authors:** Li Zhang, Yuling Wang, Ge Bai, Jianqing Zhang, Mei Yang, Xiaoli Ma

**Affiliations:** 1 Medicine VIP, First Affiliated Hospital of Xinjiang Medical University, Urumqi, 830054 China; 2 Cancer Center, First Affiliated Hospital of Xinjiang Medical University, Urumqi, 830054 China

**Keywords:** Gene, ESCC, RT-PCR, Clinicopathological features

## Abstract

The purpose of this study was to explore the relationship between the expression of vascular endothelial growth factor (VEGF), human epidermal growth factor receptor 2 (HER-2), and epidermal growth factor receptor (EGFR) mRNA in esophageal squamous cell carcinoma (ESCC) and the clinicopathological characteristics of the Han, Uyghur, and Kazakh in Xinjiang. Real-time quantitative polymerase chain reaction technology (RT-PCR) was used to detect the expression of VEGF, HER-2, and EGFR mRNA in esophageal squamous cancer tissue of 60 cases and 30 cases of VEGF, HER-2, and EGFR mRNA in esophageal cancer adjacent tissues, and analyze its relationship with clinicopathological features. The results were as follows: (1) There was no statistically significant difference in esophageal tissue VEGF, HER-2, and EGFR mRNA gene expression levels of the Han, Uyghur, and Kazakh patients (*P* > 0.05). (2) The expression levels of VEGF, HER-2, and EGFR mRNA in ESCC group were higher than those in adjacent esophageal tissue group (*P* < 0.05). (3) The expression levels of VEGF and HER-2 mRNA in ESCC of the Han patients were higher than those of the Uyghur and Kazakh patients (*P* < 0.05). (4) The expression levels of VEGF, HER-2, and EGFR mRNA in lymph node metastases were higher than those without lymph node metastasis (*P* < 0.05). (5) The expression level of HER-2 mRNA was related with the degrees of pathological differentiation, and the higher pathologic degree, the lower expression level in HER-2 mRNA (*P* < 0.05). Therefore, the following conclusions were drawn: (1) There were ethnic differences in the VEGF and HER-2 gene mRNA expression levels of the Uyghur, Han, and Kazakh patients in Xinjiang. (2) The expressions of VEGF, HER-2, and EGFR mRNA were related to the lymph node metastasis in ESCC and pathologic differentiation degree.

## Introduction

Esophageal cancer is one of the common gastrointestinal cancers in our country [1], and annually, there are about 250,000 new cases of such cancer. Xinjiang is one of the high occurrence regions of esophageal cancer, and the mortality rate accounts for 22.11 % of the total number in our country, which ranks the second [2]. Besides that, there are distinct ethnic differences in esophageal cancer in Xinjiang, and the Han and the Kazakh patients take up a large portion of the total number. In recent years, the scholars, from home and abroad, study the pathogenesis of esophageal cancer at the molecular level and achieve great clinical significance [3]. Some scholars from Xinjiang [4] have found that the changes of extracellular signal-regulated kinase (ERK)/mitogen-activated protein kinase (MAPK) signaling pathway activation level may get involved in the occurrence of Kazakh patients of early esophageal cancer by RT-PCR; as a result, nowadays, it is essential to find predict tumor markers of the prognosis of patients with esophageal cancer in Xinjiang. In this study, RT-PCR was applied to detect the expression level of gene mRNA of epidermal growth factor receptor (EGFR), vascular endothelial growth factor (VEGF), and human epidermal growth factor receptor 2 (HER-2), which are related with esophageal cancer proliferation, invasion, angiogenesis, and tumor metastasis; the relationship between the expression and the clinicopathological factors is discussed in the following text.

## Materials and methods

### Specimen source

According to the inclusion criteria of the experimental conditions, this study selected pathologic tissue specimens from 60 ESCC patients (20 cases each in Han, Uyghur, and Kazakh). Among them, there were 42 male and 18 female patients. The pathological types of the patients with Karnofsky score 0–2 were squamous cell carcinoma, tumor differentiation in grade I (poorly differentiated) by 13 cases, grade II (moderately differentiated) by 20 cases, and grade III (well differentiated) by 27 cases; there were 33 cases which had lymph node metastasis among the ESCC patients, and 27 cases without lymph node metastasis. There were 16 cases in phase II clinical stage of ESCC, 17 cases in phase III, and 27 cases in phase IV; the lesion site of 13 cases was in the upper-thoracic portion, 38 cases in the mid-thoracic portion, and 9 cases in the lower-thoracic portion. The specimens were taken from the resected ESCC pathology specimens from January 2009 to October 2011, which were preserved by the First Affiliated Hospital of Xinjiang Medical University, Department of Pathology. Thirty specimens of the tissue adjacent to carcinoma were from esophageal mucosa which is 5 cm from the tumor. And it was confirmed that esophageal tissues adjacent to cancer were normal through the pathological diagnosis, and there was no tumor invasion during the post-operation period. The specimens were reserved well, and the tissue was cut into 10 μm-thick slices, packed in 1.5 ml centrifuge tubes, each tube ranging from two to five slices. Specimens taken from patients before surgery did not receive radiotherapy and chemotherapy treatment.

### Reagents and instruments

RNA extraction reagent (TRIzol Reagent) was purchased from Beijing TIANGEN company; 2× Taq PCR Master Mix was purchased from Bo Maide biological and Fermentas company; SYBR TaKaRa Permix Ex Taq (Perfect Real Time) was purchased from TaKaRa Biotechnology (Dalian) Co., Ltd.; PCR amplification was the American BIO-RAD products; ultraviolet spectrophotometer and gel imaging systems were purchased from Shanghai Engineering Co., Ltd. in Beijing.

### Primers

Primers were synthesized by Shanghai Sangon Biological Engineering Technology Co., Ltd.

**Table:** 

Gene name	Primer name	Primer sequences (5′-3′)
β-actin	β-actin F	CCTCACCCTGAAGTACCCCA
	β-actin R	TCGTCCCAGTTGGTGACGAT
HER-2	HER-2 F	TATGCAGGGCTGACGTAGTGC
	HER-2 R	AATGTGTGCCACGAAACTGCT
EGFR	EGFR F	GCACGCCAATAGAAGG
	EGFR R	GTAAACGGCATGGCATC
VEGF	VEGF F	CACTGAGGAGTCCAACATCACC
	VEGF R	CATCTCTCCTATGTGCTGGCCT

### Experimental methods

#### The extraction of the total RNA from tissues

The extraction was performed according to the instructions: TRIzol Reagent was applied to extract RNA. The total extracted RNA was quantified, and its purity was tested by spectrophotometer, A260/A280 ≥1.8 was qualified. The integrity of the total extracted RNA was identified by agarose gel electrophoresis showing obvious 28S and 18S two bands, and then the total extracted RNA was stored at −80 °C for later use.

#### The synthesis of cDNA

1. Preparation system: RT buffer 2 ul, upstream primer 0.2 ul, downstream primer 0.2 ul, dNTP 0.1 ul, reverse transcriptase 0.5 ul, DEPC water 5 ul, RNA template 2 ul, and the total volume of 10 ul.
2. Mix the above solutions thoroughly into a tube, gently shake while flicking the tube bottom, centrifuge briefly at 6000 rpm, place it in dry bath at 65 °C for 10 min, take it out, and immediately place it on ice for 5 min. Then, successively add dNTP, RNaseA, and buffer, mix them evenly, and place it in water bath at 37 °C for 5 min, then add MTV reverse transcriptase into it at 42 °C for 60 min. After that, take it out immediately and place it in water bath at 70 °C for 10 min, then stop the reaction, and store it at −80 °C for later use.

#### Real-time PCR reactions

Take a clean 96-well plate, add 2× SYBR Green PCR Master Mix 10 ul, β-actin/EGFR/HER-2/VEGF downstream primers 1 ul each, cDNA 2 ul, and then add ddH2O 20 ul to the reaction system, after a brief centrifugation then for amplification. Reaction conditions: 95 °C for 5 min after amplification temperature 94 °C 30 s, annealing temperature (VEGF is at 57.5 °C, HER-2 is at 61.7 °C, EGFR is at 52.5 °C, and housekeeping genes are at 54.0 °C) 30 s, 72 °C 60-s cycle for 40 cycles, and finally, 72 °C extend to 10 min. Collect the fluorescence signal and analyze the melting curve; after the reaction, the quantitative result is automatically calculated by the system. Then, apply relative quantification of formula of the mRNA differential expression: the gene correction = quantitative results/housekeeping gene quantitative results, to analyze semi-quantitative.

### Statistics process

Apply SPSS 16.0 statistical software for data processing, a mean ± standard deviation for statistical description, and analyze measurement data by *t* test or analysis of variance, use the chi-square test to analyze count data, *P* ≤ 0.05 was statistically significant.

## Results

### Identification of real-time PCR system

**Fig. 1:**
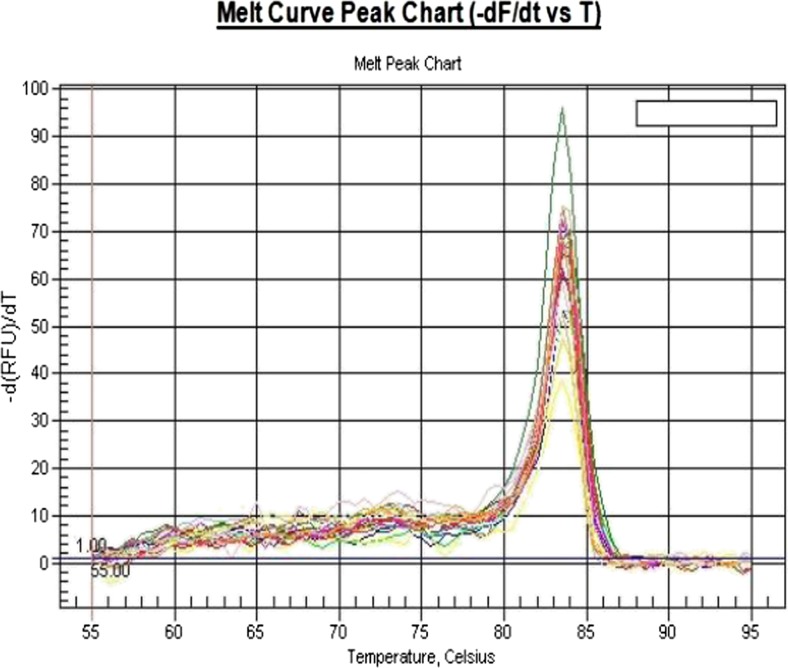
VEGF gene melting curves

**Fig. 2:**
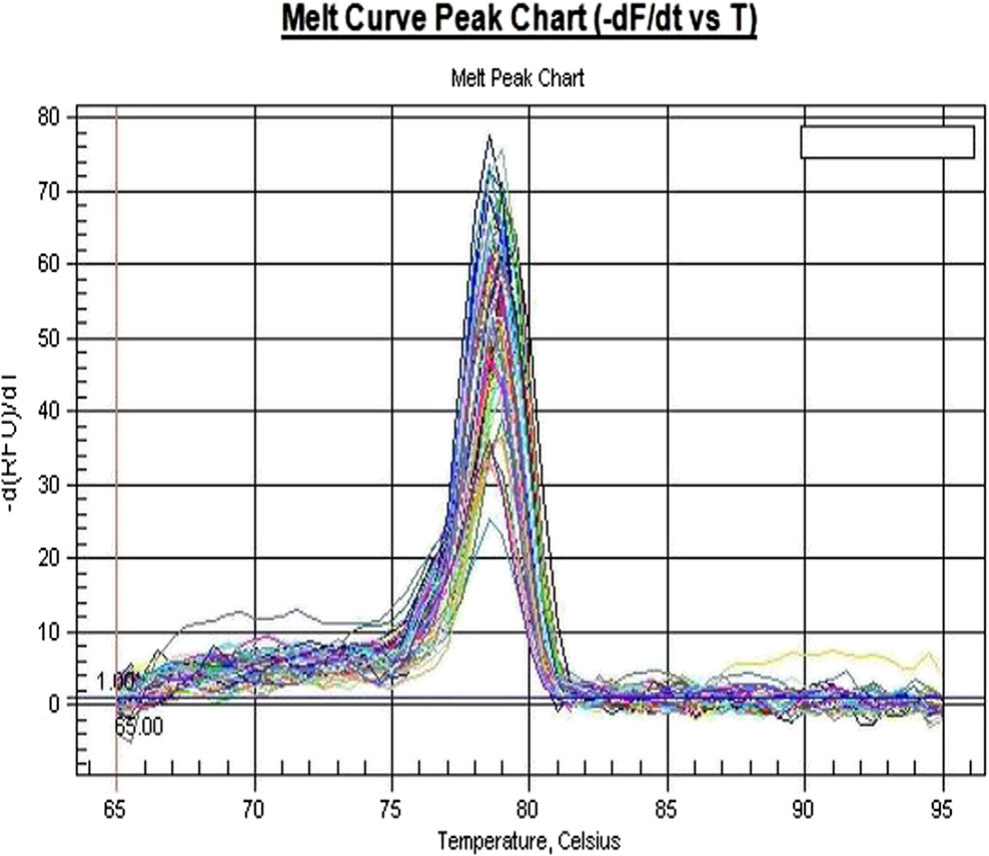
HER-2 gene melting curves

**Fig. 3:**
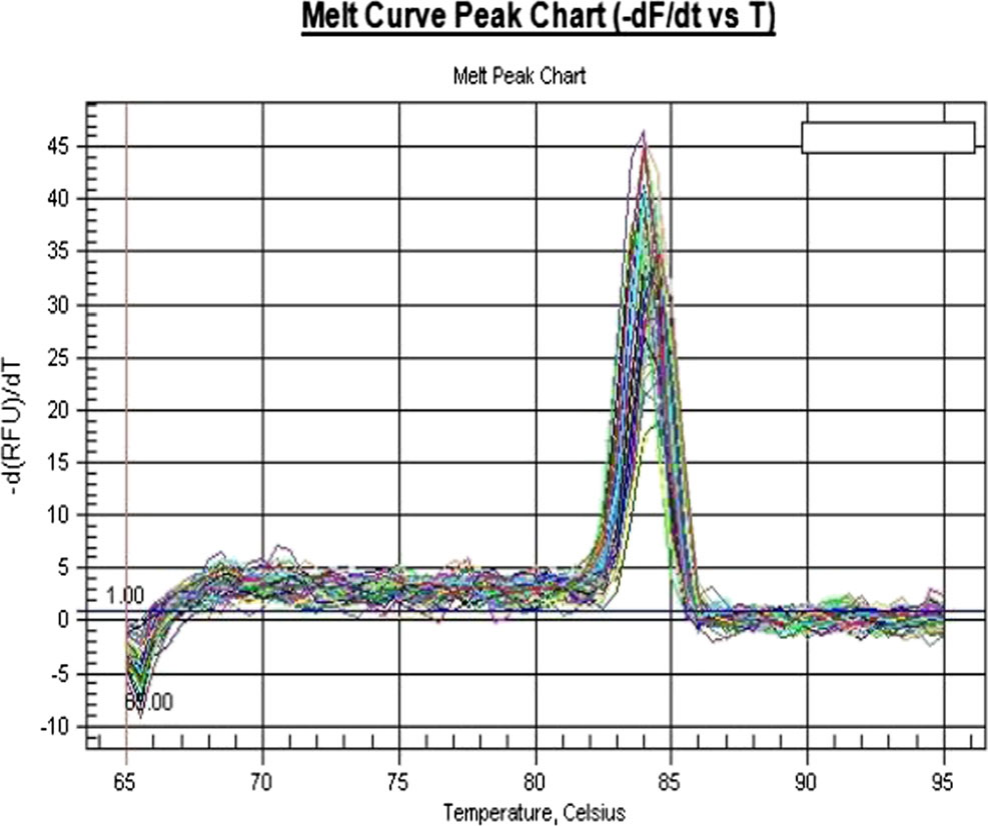
EGFR gene melting curves

**Fig. 4:**
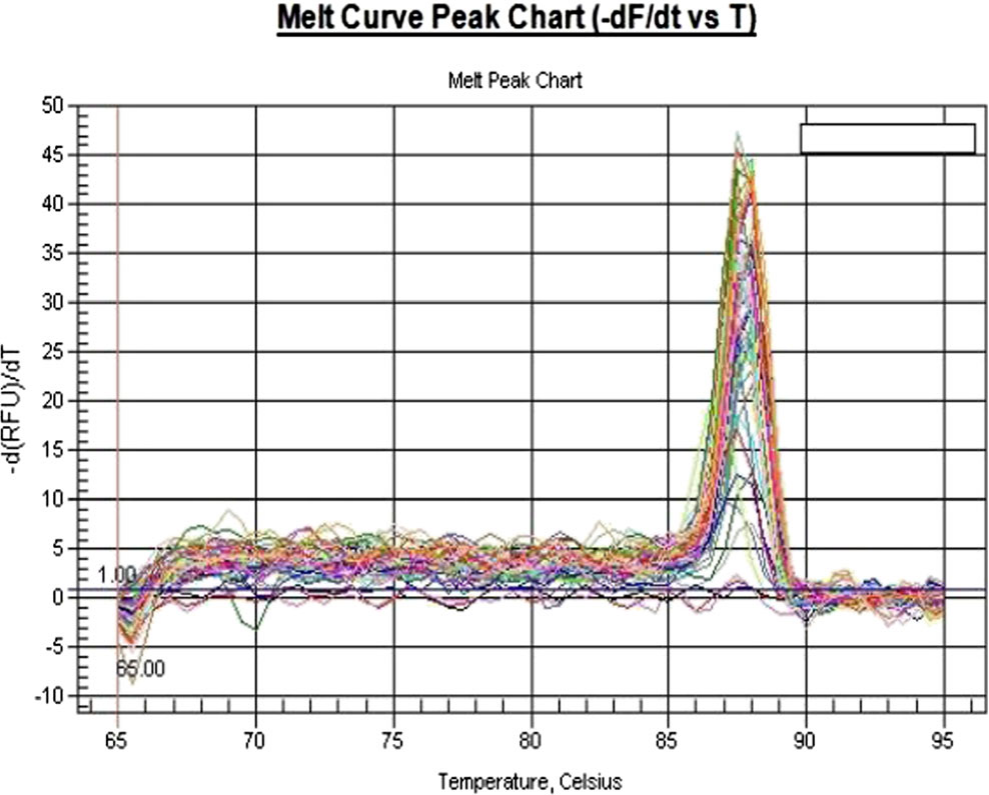
Housekeeping genes melting curves

The gene of VEGF, EGFR, and HER-2 and β-actin housekeeping gene amplification curves and the solubility curve (Figs. 1, 2, 3, and 4) indicate that PCR amplification efficiency of the target gene and the housekeeping gene is constant; rings corresponding threshold (Ct) is stable and reproducible; their specific melting curves are unimodal, primer specificity performs well, and no primer dimers. Therefore, this system can be used for quantification of the target gene.

### The comparison of VEGF, HER-2, and EGFR mRNA expression levels of the adjacent tissues to esophageal carcinoma of ESCC patients from different ethnic groups

There are no ethnic differences in the expression levels of VEGF, HER-2, and EGFR mRNA in the adjacent tissues to esophageal carcinoma (see Table 1).

**Table 1:** Comparison of VEGF, HER-2, and EGFR mRNA expression levels of the adjacent tissues to esophageal carcinoma of ESCC patients from different ethnic groups

Items	*n*	VEGF ($\overline{x}\pm s$)	*F*	*P*	HER-2 ($\overline{x}\pm s$)	*F*	*P*	EGFR ($\overline{x}\pm s$)	*F*	*P*
Han	10	0.98 ± 0.07			0.99 ± 0.09			1.08 ± 0.06		
Uyghur	10	0.94 ± 0.09	1.917	0.156	0.96 ± 0.05	0.683	0.509	1.02 ± 0.11	2.029	0.141
Kazakh	10	0.93 ± 0.09			0.97 ± 0.07			1.03 ± 0.05		

### The expression levels of VEGF, HER-2, and EGFR mRNA of ESCC and the adjacent tissues to esophageal carcinoma

The relative quantitative expression of VEGF in ESCC and the adjacent tissues to esophageal carcinoma were 1.05 ± 0.09 and 1.01 ± 0.07; the difference was statistically significant (*P* = 0.042). HER-2 relative expression levels were 0.97 ± 0.04 and 0.93 ± 0.06; the difference was statistically significant (*P* = 0.031). EGFR relative expression levels were 0.98 ± 0.47 and 0.94 ± 0.08; the difference was statistically significant (*P* = 0.045).

### The relationship between the expression levels of VEGF, HER-2, and EGFR mRNA in ESCC and clinicopathological features (see Table 2)

The expression levels of VEGF of Uyghur and Kazakh ESCC patients were 1.08 ± 0.06 and 1.05 ± 0.05. HER-2 expression levels were 0.92 ± 0.07 and 0.93 ± 0.06. The expression levels of VEGF and HER-2 of Han ESCC patients were significantly higher than those of Uyghur and Kazakh patients (*P* < 0.05) .

**Table 2:** Relationship between the expression levels of VEGF, HER-2, and EGFR mRNA in ESCC and clinicopathological features

Clinical data		*n*	VEGF ($\overline{x}\pm s$)	*t/F*	*P*	HER-2 ($\overline{x}\pm s$)	*t/F*	*P*	EGFR ($\overline{x}\pm s$)	*t/F*	*P*
Gender	Male	42	1.04 ± 0.09	−0.472	0.639	0.92 ± 0.06	−0.711	0.480	0.94 ± 0.95	−0.833	0.410
	Female	18	1.05 ± 0.07			0.94 ± 0.05			1.37 ± 2.34		
Age	>60	23	1.03 ± 0.09	−1.609	0.113	0.94 ± 0.07	0.509	0.613	1.03 ± 1.06	−0.186	0.853
	≤60	37	1.07 ± 0.07			0.93 ± 0.04			1.12 ± 1.91		
Ethnic groups	Han	20	1.08 ± 0.06	3.848*	0.027	0.99 ± 0.10	3.209*	0.048	1.50 ± 2.03	1.890*	0.067
	Uyghur	20	1.03 ± 0.06			0.92 ± 0.07			0.64 ± 0.09		
	Kazakh	20	1.05 ± 0.05			0.93 ± 0.06			1.25 ± 0.67		
Pathological grade	I	13	1.09 ± 0.07	2.029*	0.141	0.99 ± 0.11	2.521*	0.039	0.63 ± 0.07	1.832*	0.174
	II	20	1.03 ± 0.12			0.96 ± 0.07			1.69 ± 2.48		
	III	27	1.04 ± 0.06			0.94 ± 0.01			0.89 ± 0.57		
Clinical stage	II	16	0.93 ± 0.07	1.735*	0.186	1.06 ± 0.37	0.263*	0.770	0.71 ± 0.35	2.617*	0.652
	III	17	0.95 ± 0.09			1.33 ± 0.45			1.20 ± 2.10		
	IV	27	0.98 ± 0.07			1.22 ± 0.59			0.99 ± 0.97		
Lymph node metastasis	有	33	1.05 ± 0.10	2.027	0.047	1.02 ± 0.08	2.383	0.020	0.97 ± 0.04	2.198	0.031
	无	27	1.00 ± 0.07			0.97 ± 0.08			0.93 ± 0.06		

*F value

VEGF, HER-2, and EGFR mRNA expression levels in the lymph node metastasis group were 1.05 ± 0.10, 1.02 ± 0.08, and 0.97 ± 0.04, while the expression levels of the three genes without lymph node metastasis were 1.00 ± 0.07, 0.97 ± 0.08, and 0.93 ± 0.06 The expression levels with lymph node metastasis were higher than those without lymph node metastasis (*P* < 0.05).

The expression levels of HER-2 mRNA of ESCC patients in phase I was 0.99 ± 0.11, in II and III were 0.96 ± 0.07 and 0.94 ± 0.01. The HER-2 mRNA expression levels in pathological differentiation phase I, phase II, and phase III were statistically significant (*P* < 0.05), and the higher degree differentiation, the lower the expression levels of HER-2 mRNA.

The expression levels of VEGF, HER-2, and EGFR mRNA were independent of gender, age, clinical stage, and other factors.

## Discussion

Esophageal cancer is a multifactorial, a multi-stage, and multi-gene participating diseases. The amplifications and deletions of some important genes within the mucosal cells undoubtedly play a very important role in this process. The regions with a high prevalence of esophageal cancer cover the Central Asia from the former Soviet to northeastern Iran and Turkmen. Based on the studies of the population in these areas, it was found that ESCC is related with genetic factors: genetic polymorphisms can increase an individual’s susceptibility to cancer [5]. China is a high-incidence area of esophageal cancer. Among the esophageal cancer at diagnosis, there is approximately 90 % of esophageal squamous cell carcinoma [6], as a result, there may be some difference in the mechanism of the development of ESCC of our country and that of adenocarcinoma of the esophagus of Western countries [7]. Based on the results of clinical efficacy and preliminary of experimental studies, and at the same time when we retrospectively analyzed 170 cases of ESCC patients from different ethnic groups and compared the efficacy and side effects of different ethnic groups during the clinical observation, we found that the short-term efficacy of esophageal cancer chemotherapy of Han, Uyghur, and Kazakh patients in Xinjiang were similar, but the long-term survivals were different: the Han patients were better than Uyghur and Kazakh. However, Uyghur and Kazakh patients’ side effects were relatively lighter, and they were better tolerated [8]. And we simultaneously applied ELISA and immunohistochemistry to detect the expression levels of VEGF, EGFR, ES, and HER-2 and the NF-kBp in serum and tissue of Han, Kazakh, and Uyghur ESCC patients, the results indicated that there were ethnic differences in serum tumor marker levels of Han, Uyghur, and Kazakh ESCC patients in Xinjiang [9]. We believed that the fundamental reason that causes different tolerance and efficacy of the ethnic groups in Xinjiang is the polymorphism of the gene. At present, genomics technologies can help find some kind of biological markers to predict the efficacy of cancer patients, so that individualized treatment could be in the true sense.

VEGF is a specific endothelial cell-stimulating factor, and it can specifically act on endothelial cells and induce angiogenesis, increase permeability of small venules and veins, promote endothelial cell division and proliferation, and induce angiogenesis VEGF which is its major role playing in the process of tumor formation.

In this research, we found that in the adjacent tissues to esophageal carcinoma, VEGF mRNA expression levels of the Han, Uyghur, and Kazakh indicated that there was no significant statistical difference, which means that there were no ethnic characteristics in EGFR expression in the adjacent tissues to esophageal carcinoma. While the expression level of VEGF mRNA of 60 cases of ESCC patients is higher than that of the adjacent esophageal tissue, which was consistent with results of Kitadai’s [10] study: the VEGF expression levels are also different with different populations. We found that most studies indicated that the expression level of VEGF, either in protein or in serum, was closely related with clinicopathological factors. Ling Danxia et al. [11] found that the high expression of VEGF of esophageal cancer patients was related with the degree of tumor invasion and lymph node metastasis. In this study, the expression level of VEGF mRNA was closely related with ethnic and lymph node metastasis, but it was independent of gender, age, clinical stage, and degrees of pathological differentiation. This result was basically the same with Christian et al. [12] results, while the expressions of VEGF mRNA of different ethnic groups in Xinjiang were different: the expression levels of the Han ESCC patients were significantly higher than those of Uyghur and Kazakh patients. If such ethnic difference in expression levels of different ethnic groups in Xinjiang could provide the basis for clinical screening, the specificity of esophageal cancer still needs further study.

EGFR is epidermal growth factor (EGF) and proliferation signaling receptors. EGFR receptor tyrosine kinase inhibitors gefitinib and erlotinib have caused widespread concern in the clinic, and they have been applied in non-small cell lung cancer, which they inhibited EGFR autophosphorylation, thereby inhibited the signaling pathway. It has been confirmed [13] in ESCC that LRIG1 as a candidate tumor suppressor gene could feedback the high expression of inhibition of EGFR, and by increasing the high expression level of LRIG1, EGFR pathway for therapeutic intervention, therefore effectively reduces the EGFR expression levels, which provide new ideas for the clinical treatment of esophageal cancer.

Currently, the researches, from home and abroad, on the relationship between the EGFR expression levels and clinicopathological features show that abnormal expression of epidermal growth factor are closely related with the degree of tumor differentiation, sensitivity of radiotherapy and chemotherapy, tumor resistance and prognostic factors, etc. Wang [14] found in the study of esophageal adenocarcinoma that the protein expression of EGFR receptor was linked with the late clinical stage, the depth of tumor invasion, lymph node metastasis, and pathological staging; and also found that patient has a poor prognosis with positive protein expression, which can be used as a monitoring indicator of esophageal cancer prognosis. The results of this study showed that the expression levels of EGFR mRNA in ESCC was higher than that of adjacent tissues to esophageal carcinoma, and in the clinical and pathological features, the EGFR expression level was independent of gender, ethnicity, age, tumor differentiation, and clinical staging of patients with ESCC patients, but it was related with lymph node metastasis (*P* < 0.05). And some scholars believe that the length of the survival of esophageal cancer patients was independent of EGFR overexpression, or at least it could not be an independent prognostic factor [15]. As there are few relevant literature on the level of EGFR gene expression in ESCC, EGFR mRNA still cannot be an indicator of prognosis of esophageal cancer.

HER-2/neu, also known as C-erbB-2, is a member of the human epidermal growth factor receptor. There were some studies showing that HER-2 gene plays an important role in tumor carcinogenic process. HER-2/neu gene in esophageal cancer research, such as Sato-Kuwabara [16], the latest research report shows a state of HER-2 overexpression in 30.3 % of ESCC patients. In the present study, we also got the same result, the HER-2 mRNA expression levels of 60 cases of ESCC patients (0.97 ± 0.04) were higher than those of the adjacent tissues to esophageal carcinoma (0.93 ± 0.06); such difference was statistically significant.

Deilich et al. [17] found that the overexpression of HER-2 gene in ESCC was related with prognosis. And another study of ESCC of HER-2 found that the amplification of HER-2 gene in ESCC was related with the degree of tumor differentiation and staging. That means the amplification or overexpression of HER-2 gene in ESCC can be used as an independent prognostic factor. And the positive HER-2 gene expression was also considered for the purposes of neoadjuvant therapy [18]; HER-2 may be as a prognosis indicator of ESCC patients. The results of this study indicate that the expressions of HER-2 of ESCC patients were independent of gender and age, but closely related to ethnic groups, pathological grading, and lymph node metastasis of the patients. The ESCC HER-2 expression levels of Han ESCC patients were significantly higher than those of Uyghur and Kazakh patients. Differentiation and metastasis is the main factor affecting the prognosis of esophageal cancer. Therefore, the detection of expression of HER-2 in esophageal cancer in Xinjiang may have important prognostic and therapeutic significance. This also indirectly reflects that HER-2 may be one of the indicators of esophageal cancer metastasis and progression.

Currently, the targeted therapy of esophageal cancer has entered a stage of rapid development. Multi-target combination, target drugs, especially monoclonal antibody drugs combined novel cytotoxic agents, and radiotherapy are the focus of research. Although recently there are many projects of targeted therapy research, the problem is that almost all of the papers focus on the discovery of molecular markers, and few researchers further study the discovered molecular markers and apply them to clinical practice. This is the weakness of esophageal basic research, but we can enhance this point in the future. And we believe that with further research, there will be new discoveries of treatment and prevention of esophageal cancer.
